# Differential Impact of Hexuronate Regulators ExuR and UxuR on the *Escherichia coli* Proteome

**DOI:** 10.3390/ijms23158379

**Published:** 2022-07-29

**Authors:** Tatiana A. Bessonova, Maria S. Fando, Olga S. Kostareva, Maria N. Tutukina, Olga N. Ozoline, Mikhail S. Gelfand, Alexey D. Nikulin, Svetlana V. Tishchenko

**Affiliations:** 1 Institute of Cell Biophysics, Russian Academy of Sciences, PSCBR RAS, Institutskaya, 3, Pushchino 142290, Russia; tatianabessonova66@gmail.com (T.A.B.); ozoline@rambler.ru (O.N.O.); 2 Institute of Protein Research, Russian Academy of Sciences, Institutskaya, 4, Pushchino 142290, Russia; fando@vega.protres.ru (M.S.F.); kostareva@vega.protres.ru (O.S.K.); nikulin@vega.protres.ru (A.D.N.); sveta@vega.protres.ru (S.V.T.); 3 Skolkovo Institute of Science and Technology, Bolshoy Boulevard 30 Build 1, Moscow 121205, Russia; mikhail.gelfand@gmail.com; 4 Institute for Information Transmission Problems, Russian Academy of Sciences, Bolshoy Karetny Per 19 Build 1, Moscow 127051, Russia

**Keywords:** UxuR, ExuR, Ashwell pathway, *uxuAB*, *fliC*, proteome, motility, biofilm formation

## Abstract

ExuR and UxuR are paralogous proteins belonging to the GntR family of transcriptional regulators. Both are known to control hexuronic acid metabolism in a variety of Gammaproteobacteria but the relative impact of each of them is still unclear. Here, we apply 2D difference electrophoresis followed by mass-spectrometry to characterise the changes in the *Escherichia coli* proteome in response to a *uxuR* or *exuR* deletion. Our data clearly show that the effects are different: deletion of *uxuR* resulted in strongly enhanced expression of D-mannonate dehydratase UxuA and flagellar protein FliC, and in a reduced amount of outer membrane porin OmpF, while the absence of ExuR did not significantly alter the spectrum of detected proteins. Consequently, the physiological roles of proteins predicted as homologs seem to be far from identical. Effects of *uxuR* deletion were largely dependent on the cultivation conditions: during growth with glucose, UxuA and FliC were dramatically altered, while during growth with glucuronate, activation of both was not so prominent. During the growth with glucose, maximal activation was detected for FliC. This was further confirmed by expression analysis and physiological tests, thus suggesting the involvement of UxuR in the regulation of bacterial motility and biofilm formation.

## 1. Introduction

Transcription factors control many steps of a bacterial life cycle and operate most metabolic switches that take place in *Escherichia coli*. In *E. coli* and closely related proteobacteria, hexuronic acids are utilised via the Ashwell pathway mediated by the two local regulators, UxuR and ExuR. These proteins are paralogous, as the *exuR* gene resulted from duplication of *uxuR* [1]. Together, they control genes encoding all key enzymes for hexuronate utilisation, specifically, oxidoreductase UxuB and mannonate dehydratase UxuA, glucuronidase UidA, glucuronate/galacturonate isomerase UxaC, hexuronate transporters ExuT and GntP, and glucuronide transporters UidB and UidC. Moreover, both regulators serve as autorepressors [1,2,3,4,5,6,7,8,9,10,11,12]. Initially, UxuR was believed to be responsible for the expression of all genes coding for enzymes and transporters related to glucuronate metabolism [5,7,9], while ExuR—for galacturonate metabolism [2,3,6,7]. Later, ExuR and UxuR were shown to cross-regulate each other’s targets, probably forming heterodimers [10,11]. Interchangeability of D-galacturonate and D-glucuronate as effectors and heterodimer formation was further confirmed in [12]. As such, we proposed that the role of ExuR might be in the depression of UxuR-repressed genes via the formation of the UxuR-ExuR heterodimer [12].

Several studies suggested that hexuronate metabolism could be important for the ability of proteobacteria to move and to colonise a host intestine [13,14,15]. Virulence and intestinal colonisation of *Citrobacter rodentium* and enterohaemorrhagic *E. coli* (EHEC) are regulated by the diet-derived galacturonic acid acting via ExuR [16]. However, there is no direct evidence of the involvement of UxuR, a key regulator of hexuronate metabolism in *E. coli* K-12 MG1655, in the modulation of bacterial motility and colonisation.

Modern transcriptomics can identify RNA-targets of the studied transcription factors, but the resulting levels of proteins may be not proportional to concentrations of the respective mRNAs due to, e.g., differences in the translation rates or protein degradation. Thus, proteomics approaches, in addition to other omics, are useful to evaluate cellular response to deletion of a particular gene, or to a change in growth conditions (for example, a different carbon source). The most popular way to compare proteome maps of the two strains or one strain grown in two different conditions is two-dimensional difference gel electrophoresis (2D DIGE or DIGE), a modification of the 2D electrophoresis where two or three samples are compared on one gel, using varieties of cian dyes that attach covalently to free amino groups of lysines. This improves the reproducibility of experimental data and simplifies identifying differences in the studied proteomic maps.

Here, we use DIGE to investigate changes occurring in the *E. coli* proteomes in response to a deletion of genes encoding regulators of hexuronate metabolism, ExuR, and UxuR during growth on different carbon sources. To prove the detected effects, we test the expression of genes encoding the most influenced proteins and check the ability of the wild type and its *uxuR* deletion derivative to move and to form biofilms.

## 2. Results

### 2.1. exuR Deletion Did Not Influence the Proteome of E. coli K-12 MG1655

**Figure 1:**
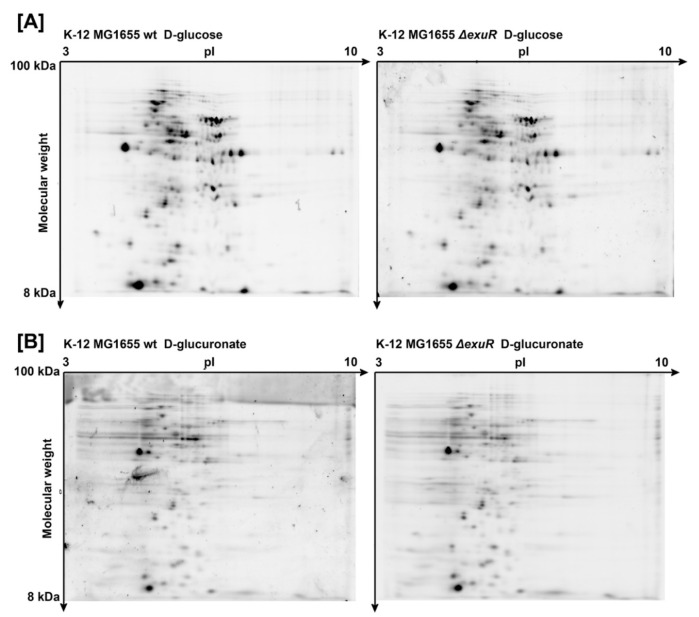
Proteomic maps of the wild type *E. coli* K-12 MG1655 and *E. coli* K-12 MG1655 Δ*exuR* during growth with D-glucose (**A**) and D-glucuronate (**B**). For each condition, 218 (**A**) and 160 (**B**) proteins were detected in both strains.

Surprisingly, the proteomic maps of the wild type *E. coli* K-12 MG1655 and its Δ*exuR* derivative did not differ in the spectrum of proteins, both in the cells growing with D-glucose (Figure 1A) and with D-glucuronate (Figure 1B).

This was from one side rather unexpected since ExuR was predicted as a regulator of hexuronate metabolism with a function similar to that of UxuR [1,7]. On the other, it is in agreement with our previous qRT-PCR and β-galactosidase data where the effects of *exuR* deletion on its predicted targets were moderate [12].

### 2.2. Effects of uxuR Deletion on the Proteome of E. coli K-12 MG1655

**Figure 2:**
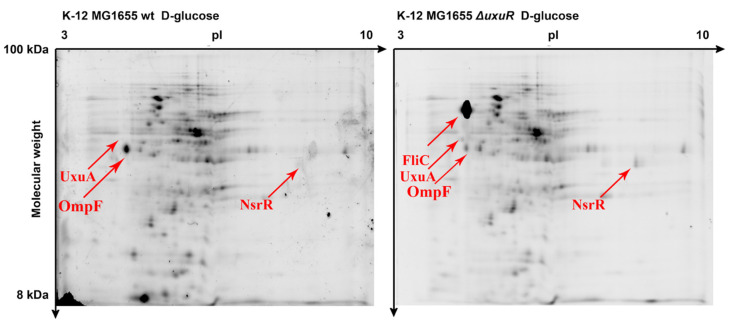
Proteomic maps of the wild type *E. coli* K-12 MG1655 and *E. coli* K-12 MG1655 Δ*uxuR* during growth with D-glucose. For D-glucuronate, gels are shown in Figure S2. Two hundred and eighteen proteins were detected in both strains.

Unlike the previous case, the comparison of the proteomic maps of the wild type *E. coli* K-12 MG1655 and *E. coli* K-12 MG1655 Δ*uxuR* revealed several proteins with significantly changed levels. Rather predictably, these proteins were different during the growth with D-glucose (Figure 2 and Table 1) and D-glucuronate (Figure S2 and Table 2).

Figure 2 demonstrates that the proteins most strongly affected by the *uxuR* deletion are D-mannonate dehydratase UxuA, the key enzyme of the Ashwell pathway, and a structural protein of bacterial flagella FliC. The latter showed a dramatic increase suggesting UxuR involvement in the regulation of bacterial motility. Changes in the concentration of these proteins during growth with D-glucuronate were much less dramatic (Supplementary Figure S2 and Table 2), which could be explained by the derepression of the UxuR-mediated genes by ExuR-UxuR heterodimers in the presence of this substrate. Porin OmpF was inhibited in the *uxuR* deletion derivative independent of the carbon source.

This might indicate that UxuR plays a role in the constitutive regulation of this transport system. Other proteins with altered levels were related to several metabolic pathways, and, interestingly, to nitrogen metabolism, as UxuR repressed expression of the transcriptional regulator NsrR. Changes in the protein spectrum of the wild type cells during growth with glucuronate are shown in Supplementary Figure S3 and Table S1. In brief, all major changes were predictably related to metabolic enzymes and transporters, as well as proteins related to nitrosative and osmotic stress, probably reflecting stress conditions induced by a very poor medium.

### 2.3. Effects of uxuR Deletion on Motility of E. coli K-12 MG1655 

**Figure 3:**
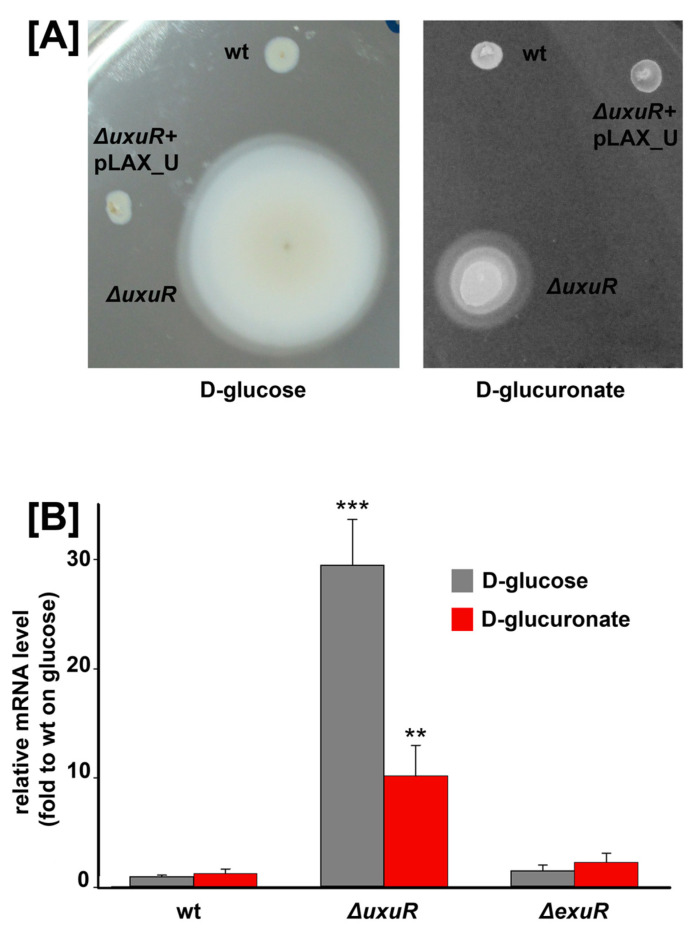
(**A**) Bacterial motility assay in 0.3% agar in the presence of 0.2% D-glucose or D-glucuronate. (**B**) Effects of UxuR and ExuR on *fliC* transcription. A panel represents qRT-PCR data for the *E. coli* K-12 MG1655 strain and its *uxuR* or *exuR* deletion mutants. No changes were detected in the *hns*-mRNA levels that were used as controls. mRNA levels are expressed relative to the parent strain during the growth on glucose. Error bars represent the standard deviations from four biological replicates. Significant differences were defined by *p* < 0.01 (**) and *p* < 0.001 (***) compared to the wild type strain during growth with glucose.

To check whether UxuR has an effect on bacterial motility, we measured the mobility zones of the wild type strain and its Δ*uxuR* derivative during growth with the addition of D-glucose or D-glucuronate (Figure 3A).

After 6 h of growth at 37 °C, the motility zones were much larger in the *uxuR* mutant, and this effect was less pronounced in the presence of D-glucuronate. This observation is in line with the DIGE result and suggests that UxuR controls *E. coli* motility in an effector-dependent manner.

To further check this assumption, we measured the expression of *fliC* in the wild type K-12 MG1655 and its *exuR* and *uxuR* deletion mutants during growth on D-glucose or D-glucuronate as a sole carbon source. Figure 3B shows that indeed *uxuR* deletion resulted in an approximately 30-fold activation of *fliC* during growth with glucose. D-glucuronate impaired this effect, and *exuR* deletion led to only insignificant changes in the *fliC* expression.

### 2.4. Effects of uxuR Deletion on the Biofilm Formation of E. coli K-12 MG1655

**Figure 4:**
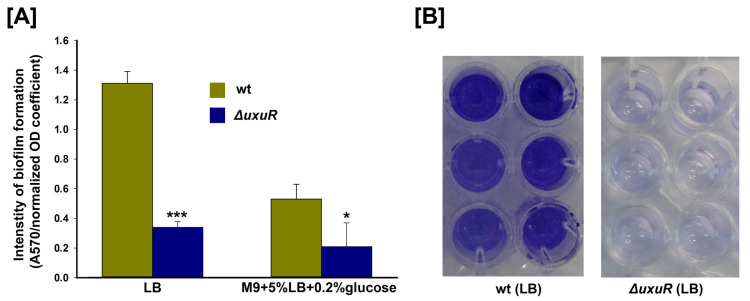
(**A**) Relative efficiency of biofilm formation of the wild type *E. coli* K-12 MG1655 strain and its *uxuR* deletion mutant after 48 h of microaerobic growth on LB or M9 medium. (**B**) Example of the staining intensity after 48 h of microaerobic growth on LB. Error bars represent the standard deviations from six biological replicates. Significant differences were defined by *p* < 0.05 (*) and *p* < 0.001 (***) compared to the wild type strain.

Inhibition of bacterial motility by UxuR highlights the importance of the Ashwell pathway for the colonisation of host organisms. Colonisation processes are normally accompanied by the formation of bacterial biofilms that help bacteria to survive in harsh conditions. Figure 4 illustrates the influence of *uxuR* deletion on the relative intensity of biofilm formation. As expected, UxuR is necessary for the effective generation of biofilms, especially during growth on rich LB medium (Figure 4A,B).

During growth on a minimal medium with glucose, the effect of UxuR was much less statistically significant (Figure 4A). We observed that even on one plate two neighbouring wells can yield opposite results, and even the descendants of one colony may show different behaviour (Supplementary Figure S4). We saw the same pattern on DIGE, as in some experiments no FliC activation was detected (an example is shown in Supplementary Figure S1). This might hint at an additional level of regulation that bacteria switch on in poor conditions to enhance the survival rate, and this is a subject for our next study.

## 3. Discussion

One of the main findings of this study is the globally different effects of ExuR and UxuR earlier predicted to have similar functions, on the proteomes of *E. coli*. The absence of significant changes in the spectrum of proteins in the *exuR* deletion mutant in both tested conditions, typically representing the *E. coli* metabolic lifestyles, might suggest that this regulator does not play a major role in the control of the Ashwell pathway, as it was assumed previously, at least, as a self-sufficient protein.

On the other hand, its role was found to be rather uncertain already in the 1980s [11], and in line with this, we previously showed that *uxuR* and *exuR* deletions led to different and even opposite effects on bacterial growth in the presence of D-glucuronate [12]. Deletion of *uxuR* resulted in dramatic inhibition of growth, confirming the role of UxuR as an essential regulator of hexuronate metabolism that cannot normally function in its absence. Deletion of *exuR*, in contrast, led to a slightly enhanced growth of bacteria compared to the wild type. This minor physiological dependence is consistent with the moderate effect of ExuR removal on the expression of its predicted targets revealed by gRT-PCR and β-galactosidase testing [12]. Thus, although our data do not exclude the specific involvement of ExuR or other products from the same genomic locus in the fine-tuning of the cellular transcriptome, based on the fact that the proteomic maps of the *exuR* mutant were not distinguishable from wild-type maps, it seems that ExuR itself does not significantly affect the expression of at least metabolically important genes.

**Figure 5:**
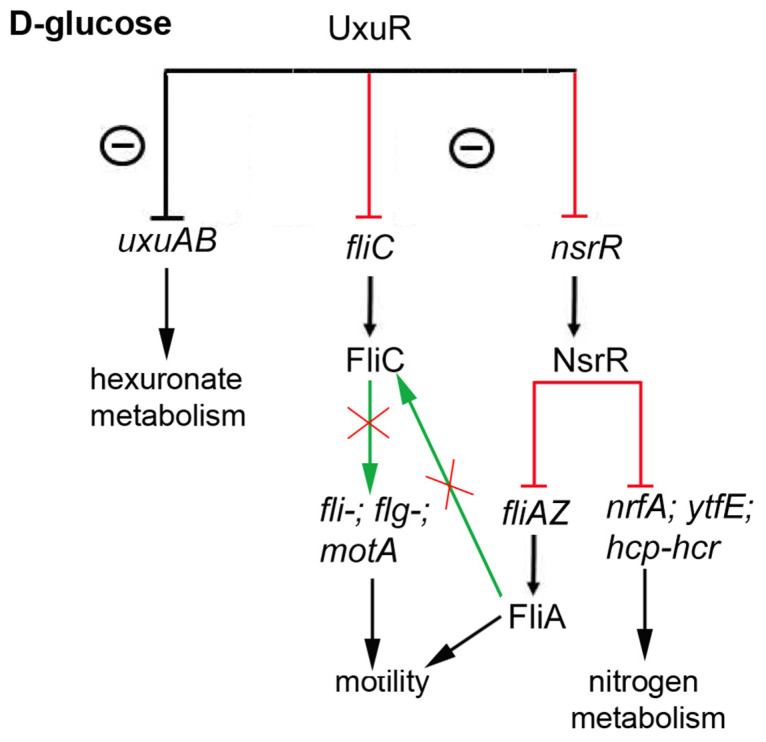
Effects of UxuR on different processes in *E. coli* during growth with D-glucose, based on the proteomic analysis. Activation is shown in green, inhibition—in red.

In contrast, deletion of *uxuR* significantly altered the spectra of detected proteins during the *E. coli* growth both on glucose, a universal carbon source, and on glucuronate, the substrate, and the intermediate of the Ashwell and Entner-Doudoroff pathways, but the changes were different. In the presence of glucose, the enzymes of sugar metabolism were affected, as well as proteins related to virulence and flagellar motion [17,18]. In particular, significant activation of the structural flagellar protein FliC had been registered for the first time (Figure 1, Figure 3, and Figure 5, Table 1), confirming the importance of UxuR for the control of the *E. coli* motility. Persistence of *E. coli* in the host organism suggests colonisation of epithelial cells in the intestine that is consistently reduced with activation of bacterial motility and is dependent on hexuronates that are controlled by UxuR [14,15]. During growth with D-glucuronate, when metabolism mainly goes via the Ashwell pathway, no such effect had been observed (Table 2, Figure 3), in agreement with the improved ability of bacteria to colonise the intestine [14,15,16].

An enhanced level of mannonate dehydratase synthesis (encoded by *uxuA*) in the K-12 MG1655 Δ*uxuR* can be considered as expected being explained by the loss of the UxuR repression at the *uxuAB* promoter [19]. The direct effect of UxuR on the *uxuAB* promoter was confirmed by measuring the β-galactosidase activity of the pRW224-uxuAB plasmid in the wild type strain, its Δ*uxuR* derivative, and the mutant complemented with the plasmid producing UxuR at the physiological level (Figure S5).

Increased level of the NsrR transcription factor was far less predictable (Table 1). Although the difference between the strains was not very significant (score of 40), it deserves some attention because transcription factors are normally present in the cells in small amounts and thus are poorly detected in the proteomes. The *nsrR* gene does not belong to the UxuR regulon which is rather small [20,21]. However, in many strains of *Salmonella enterica*, the gene coding for the HCP protein is located in close proximity to *uxuR* (microbesonline.org), which assumes a possible involvement of UxuR in the regulation of nitrogen metabolism (Figure 5). NsrR, in contrast, controls the expression of 34 operons of different functionality, including five local (*dsdC*, *feaR*, *fhlA*, *norR*, *pdhR*) and at least one global (*lrp*) transcriptional regulator. According to the information in RegulonDB [20], the strongest inhibitory effect of NsrR was detected for just four genomic loci; among them, two operons of nitrogen assimilation (*nrfABCDEFG* and *hcp-hcr-poxB-ltaE-ybjT*), the *ytfE* gene with mutations leading to a sensitivity of *E. coli* to nitric oxide [22,23], and the *fliAZ-tcyJ* operon. *fliA* encodes for a σ-factor, controlling all flagellar genes, including *fliC*. Due to that, the enhanced levels of NsrR should inhibit transcription from *fliA* and, hence, *fliC*. However, we observed the opposite effect. This might indicate that NsrR participates in flagellin biogenesis, sustaining its production at an optimal level. However, the question about molecular mechanisms leading to the accumulation of FliC in the mutant cells remains open. Another interesting question that still needs an answer is how a bacterial cell with the *uxuR* deletion decides whether to form a biofilm or not.

Metabolic networks in living organisms, even prokaryotic, are complex systems of biochemical reactions being regulated mostly by the physical and chemical conditions in the environment. From this point of view, carbon sources serve not only as a source of nutrition but also play key roles in bacterial adaptation to environmental conditions. In the presence of D-glucuronate, metabolic switches led to changes in the synthesis of hemotaxis-related D-galactose-binding periplasmic protein MglB and Colicin I receptor CirA (Table 2). The latter is a membrane receptor protein interacting with antimicrobial colicines 1A and 1B; receptors of this type may also participate in iron transport [24]. At present, we cannot provide a biological explanation for its inhibition; however, taking into account changes in the production of two other transport-related proteins, HisJ and OmpF, we might assume that deficiency of nutrients in the media pushes on the synthesis of one transport protein and switches off others, less needed in the current conditions. In line with this assumption, some enzymes were activated (such as L-malate-hydrolyase FumA) and others were inhibited (2-iminobutanoate/2-iminopropanoate deaminase RidA) (Table 2).

In summary, the comparative analysis of proteomic maps of the wild type *E. coli* K-12 MG1655 strain and its *uxuR* and *exuR* mutant derivatives suggest differential roles of their protein products in the regulation of gene expression and, specifically, a complex network for UxuR. This transcription factor, or any other products being synthesized from its gene, may affect not only the production of enzymes and transporters of sugar metabolism but also bacterial motility, biofilm formation, and, indirectly, iron transport and nitrogen metabolism.

## 4. Materials and Methods

### 4.1. Bacterial Strains and Cultivation Conditions

*Escherichia coli* K-12 MG1655 wild type strain and its derivatives with deleted *uxuR* and *exuR* genes were used for all experiments. The genes were disrupted in *E. coli* BW25113 using recombineering technique [25], and then the gene::kan mutations were transferred by P1-transduction into *E. coli* K-12 MG1655 and into its Δ*lac* derivative, *E. coli* M182 [12]. To complement the *uxuR* deletion, the pLAX_U plasmid was constructed based on the pLAX185 [26] that allows recombinant gene expression directly in the parent strain.

To prepare samples for DIGE, 1 mL of overnight culture grown in LB was transferred to 250 mL of M9 medium containing 5% LB and 0.2% D-glucose or D-glucuronic acid as a primary carbon source and was then cultivated at 37 °C for 4.5 h (OD_650_ = 0.3–0.4) under constant shaking. The cells were stopped with centrifugation at 5000× *g*, 4 °C; for 10 min.

### 4.2. Preparation of Samples for DIGE 

Cell pellets from 20 mL of culture were resuspended in 3 mL of buffer containing 2% Triton-X-100, 65 mM DTT, 10 mM EDTA, 20 mM Tris-HCl pH 8.0, and 15 mg of Protease Inhibitor Mini Tablets, EDTA-free (Thermo Fisher Scientific, Waltham, MA, USA). Cells were disrupted on the Sonic; Dismembrator 550 (Thermo Fisher Scientific, Waltham, MA, USA) for 15 min at 35% intensity. Cell debris and ribosomal fraction were precipitated by ultracentrifugation at 213,000× *g*, 4 °C for 30 min. Proteins were precipitated with the addition of 1:1 (V:V) of 20% TCA and 0.2% DTT in ice-cold acetone to supernatant, and the resulting mixture was then incubated at 4 °C overnight. Denatured proteins were centrifuged at 20,000× *g*, 4 °C for 15 min. The pellet was washed 3 times with 0.2% DTT in ice-cold acetone followed by 1 wash with Milli-Q water, air dried, and dissolved in 40 µL of buffer containing 7 M urea, 2 M thiourea, 4% (W:V) CHAPS, 65 mM DTT, 20 mM Tris-HCl, pH 8.5. Protein concentration was measured using the Quick Start™ Bradford Protein Assay Kit 1 (Bio-Rad, Berkeley, CA, USA) and validated using SDS-PAGE.

### 4.3. Protein Labeling

Samples were visualised using Lumiprobe 3Dye™ (Moscow, Russia). Dyes were dissolved in 98% dimethylformamide to 1 mM concentration. A protein sample was stained with either Cy3 or Cy5 (0.4 mM final concentration) and incubated in the dark at 4 °C for 30 min. The staining reaction was stopped by adding 1 µL of 10 mM lysine followed by 10 min incubation at 4 °C in the dark. Then, the buffer containing 7 M urea, 2 M thiourea, 4% (W:V) CHAPS, 0.6% (V:V) ampholytes, and 65 mM DTT, was added to a final volume of 330 µL. Proteins isolated from samples to be compared were mixed and used for the 2D electrophoresis.

### 4.4. 2D Differential Electrophoresis (DIGE) 

Isoelectric focusing of proteins was made on the Protean i12 IEF system (Bio-Rad, Berkeley, CA, USA) using commercial 17 cm gels with the 3–10 pH gradient (pH 3–10 IPG strip, Bio-Rad, Berkeley, CA, USA). The programme used for isoelectric focusing is shown in Table 3.

Separation of proteins in the second direction according to their molecular weights was made using SDS-PAGE (gel size 18.5 × 20 cm). After isoelectrofocusing, the IPG strip was incubated in the buffer containing 6 M urea, 2% SDS, 20% glycerol, 10 mM DTT, 0.5 M Tris-HCl, pH 8.8, followed by a 20 min incubation in the same solution supplemented with 10 mM iodoacetamide.

Electrophoresis was performed on the PROTEAN II xi 2-D Cell system (Bio-Rad, Berkeley, CA, USA) using the Laemmli protocol [27]. Concentrating gel contained 4% acrylamide (acrylamide:methylene bis acrylamide = 37.5:1), 125 mM Tris-HCl, pH 6.8, 0.1% SDS, 0.2% (V:V) TEMED, and 0.1% (V:V) APS. Resolving gel contained 15% acrylamide (acrylamide:methylene bis acrylamide = 37.5:1), 375 mM Tris-HCl, pH 8.8, 0.1% SDS, 0.2% (V:V) TEMED, and 0.1% (V:V) APS. Gels were run in the standard tris-glycine buffer (25 mM Tris-HCl, pH 8.3, 0.192 M glycine, 0.1% SDS).

Proteins were visualized using the ChemiDoc MP Imaging System (Bio-Rad, Berkeley, CA, USA) and Image Lab (Bio-Rad, Berkeley, CA, USA). Proteomes of the cells growing on different carbon sources or of the wild type and mutant cells were compared on one gel. All experiments were made at least in duplicates. For the *uxuR* deletion mutant, the experiment was repeated four times.

### 4.5. MALDI-TOF Mass-Spectrometry 

Proteins were extracted from the gel in 150 μL 50% (V:V) acetone and 50% (V:V) 50 mM ammonium bicarbonate at 37 °C (two times for 15 min) followed by dehydration in 100% acetonitrile. Then, 25 ng trypsin in 50 mM ammonium bicarbonate buffer was added, and the samples were incubated at 37 °C for 16 h. Then, the supernatant was transferred to new tubes, and hydrolysate was either mixed 1:1 (V:V) with a solution containing 2% 2,5-dihydroxybenzoic acid in 50% acetonitrile and 3% trifluoroacetic acid or mixed with 400 μL of 60% acetonitrile in 0.1% trifluoroacetic acid, vacuum dried at 45 °C, and dissolved in 20 μL of 30% acetonitrile in 0.1% trifluoroacetic acid.

Analysis was made on a MALDI-TOF/TOF (Ultraflex, Bruker Daltonics, Bremen, Germany) equipped with the Nd:YAG laser in a reflector mode, *m*/*z* of 700–3500, 50 ppm (“Human Proteome” Facility) [28], or on a MALDI-TOF / TOF (Rapiflex, Bruker Daltonics, Bremen, Germany) equipped with the SmartBeam III laser in a reflector mode, *m*/*z* of 600–5000, 50 ppm (Skoltech Core Proteomics Facility). The spectrometer was calibrated with the Peptide Calibration Standard II and, additionally, by the trypsin autolysis peaks.

Proteins were identified using MASCOT (www.matrixscience.com, last accessed on 24 April 2022) [29] based on the SwissProt database (www.uniprot.org, last accessed on 24 April 2022). Candidate proteins were considered confident if the score was higher than 49 (*p* < 0.05). All experiments were made at least in duplicates.

### 4.6. Biofilm and Motility Assays

To measure the intensity of biofilm formation, 10 μL of an overnight culture was used to inoculate a 96-well microtiter plate (REF 83.3924, Sarstedt, Nümbrecht, Germany) containing 90 μL of LB or M9 + 5% LB. The plate was incubated for 48 h at 37 °C in microaerobic conditions without shaking. Then, unattached cells were washed with phosphate buffer saline (PBS), while the remaining were fixed, dried, and stained with 1% crystal violet as described in [30]. Each experiment was made at least with three independent cultures in triplicate on each plate.

For motility assays, bacterial strains were grown in LB until the late-log phase, and then one microliter was placed onto an LB plate containing 0.3% agar (swimming motility plate). The plates were incubated at 37 °C for 4–6 h and motility zones from the point of inoculation were examined [31].

### 4.7. RNA Extraction and Quantitative PCR 

RNA was extracted using the TriZol reagent (Thermo Fisher Scientific, Waltham, MA, USA) according to the manufacturer’s protocol and treated with the DNAse I (New England Biolabs, Ipswich, MA, USA) for 1 h at 37 °C followed by 10 min of inactivation at 70 °C. RNA concentration was measured on a NanoDrop-1000 spectrometer (Thermo Fisher Scientific, Waltham, MA, USA), and quality was checked on a 4% polyacrylamide gel with 8 M urea. Reverse transcription was made with the RevertAid M-MulV reverse transcriptase (Thermo Fisher Scientific, Waltham, MA, USA) and a gene-specific primer fliC-R 5′-TTAGTACCGGTAGTGGCCTG-3′. Briefly, 1 μg of RNA was mixed with 4 pmol of the primer and heated for 10 min at 70 °C. Then, 8 μL of the preheated master mix was added containing 1× buffer (Thermo Fisher Scientific, Waltham, MA, USA) and 2 μL dNTP mix (2.5 mM each). The samples were chilled on ice for 2 min, then 40 units of the enzyme were added, and the mixture was further incubated for 40 min at 39 °C. The reaction was stopped at 85 °C for 5 min.

A DT-Lite thermocycler (DNA-Technology, Protvino, Russia) and qPCR-HS mix (Evrogen, Moscow, Russia) were used for quantitative PCR (qRT-PCR). The second primer was fliC-F 5-TCTGTCTTCTGGCTTGCGTA-3′. The *hns* gene coding for the nucleoid protein was used as a normalization control [12]. No PCR products were detected in negative controls in the absence of reverse transcriptase. Data obtained from at least three biological samples and analyzed in three statistical replicates were calculated by the ΔC_t_ method. The error bars indicate the standard deviations of corresponding mean values.

### 4.8. β-Galactosidase Assays

To assay transcription initiation from the *uxuAB* promoter, corresponding DNA fragments were cloned into the *lac* expression vector pRW224 using EcoRI and HindIII sites, and β-galactosidase activities in the *E. coli* strain M182 (Δ*lacZ* derivative of K-12 MG1655) carrying these recombinant plasmids were measured as described in [32]. Activity was calculated as nanomoles of ortho-nitrophenyl-β-galactoside (ONPG) hydrolysed per min per mg of dry cell mass. Results reported are the means of at least three biological replicates each analysed in two assay repeats. The error bars are the standard deviations of all assays.

## Figures and Tables

**Table 1:** Proteins with changed levels in the *E. coli* K-12 MG1655 ΔuxuR as compared to the wild type strain during growth on minimal medium with D-glucose as the main carbon source. Proteins with statistically significant scores are in bold. All proteins that were cut and analysed are shown in Supplementary Figure S1 and all proteins identified are listed in Supplementary Table S1.

№	Protein	Score	Function	*M*_w_, Da	Effect of *uxuR* Deletion
1	FliC	247	**Flagellar filament structural protein**	51,265	activated
2	UxuA	75	**D-Mannonate dehydratase**	44,838	activated
3	GrcA	51	**Stress-induced alternate pyruvate formate-lyase subunit**	14,284	inhibited
4	YeiG	49	**S-formylglutathione hydrolase**	31,308	inhibited
5	OmpF	48	Outer membrane porin F	39,333	inhibited
6	NsrR	40	HTH-type transcriptional repressor	15,583	activated
7	YdbK	33	Pyruvate-flavodoxin oxidoreductase	128,743	inhibited
8	EutM	27	Putative ethanolamine catabolic microcompartment shell protein	9859	inhibited

**Table 2:** Proteins with changed levels in the *E. coli* K-12 MG1655 ΔuxuR as compared to the wild type strain during growth on minimal medium with D-glucuronate as the main carbon source. Proteins with statistically significant scores are in bold. All proteins that were cut and analysed are shown in Supplementary Figure S2 and all proteins identified are listed in Supplementary Table S1.

№	Protein	Score	Function	*M*_w_, Da	Effect of *uxuR* Deletion
1	CirA	202	**Colicin I receptor**	73,850	inhibited
2	MglB	140	**D-galactose-binding periplasmic protein**	35,690	activated
3	FumA	82	**Fumarate hydratase class I, aerobic**	60,260	activated
4	HisJ	73	**Histidine-binding periplasmic protein**	28,466	activated
5	RidA	70	**2-iminobutanoate/2-iminopropanoate deaminase**	13,603	inhibited
6	OmpF	48	Outer membrane porin F	39,333	inhibited
7	GrcA	39	Stress-induced alternate pyruvate formate-lyase subunit	14,259	inhibited

**Table 3:** Sequential separation of protein samples in the first direction.

	Voltage, V.	Time, min.
1	100	120
2	250	30
3	10,000	120
4	10,000	43,000 Volt-time *

* Volt-time is an integral of the voltage applied at the time of sample separation.

## Data Availability

Not applicable.

## Supplementary Materials

The following supporting information can be downloaded at: https://www.mdpi.com/article/10.3390/ijms23158379/s1.
