# Supplementary material for: Differential Impact of Hexuronate Regulators ExuR and UxuR on the Escherichia coli Proteome
Source: Int J Mol Sci. 2022 Jul 29;23(15):8379. doi: 10.3390/ijms23158379 (PMC9369180; doi:10.3390/ijms23158379)
Supplement: Supplementary file 1 [file ijms-23-08379-s001.zip › Supplementary figures with legends_rev.pdf]

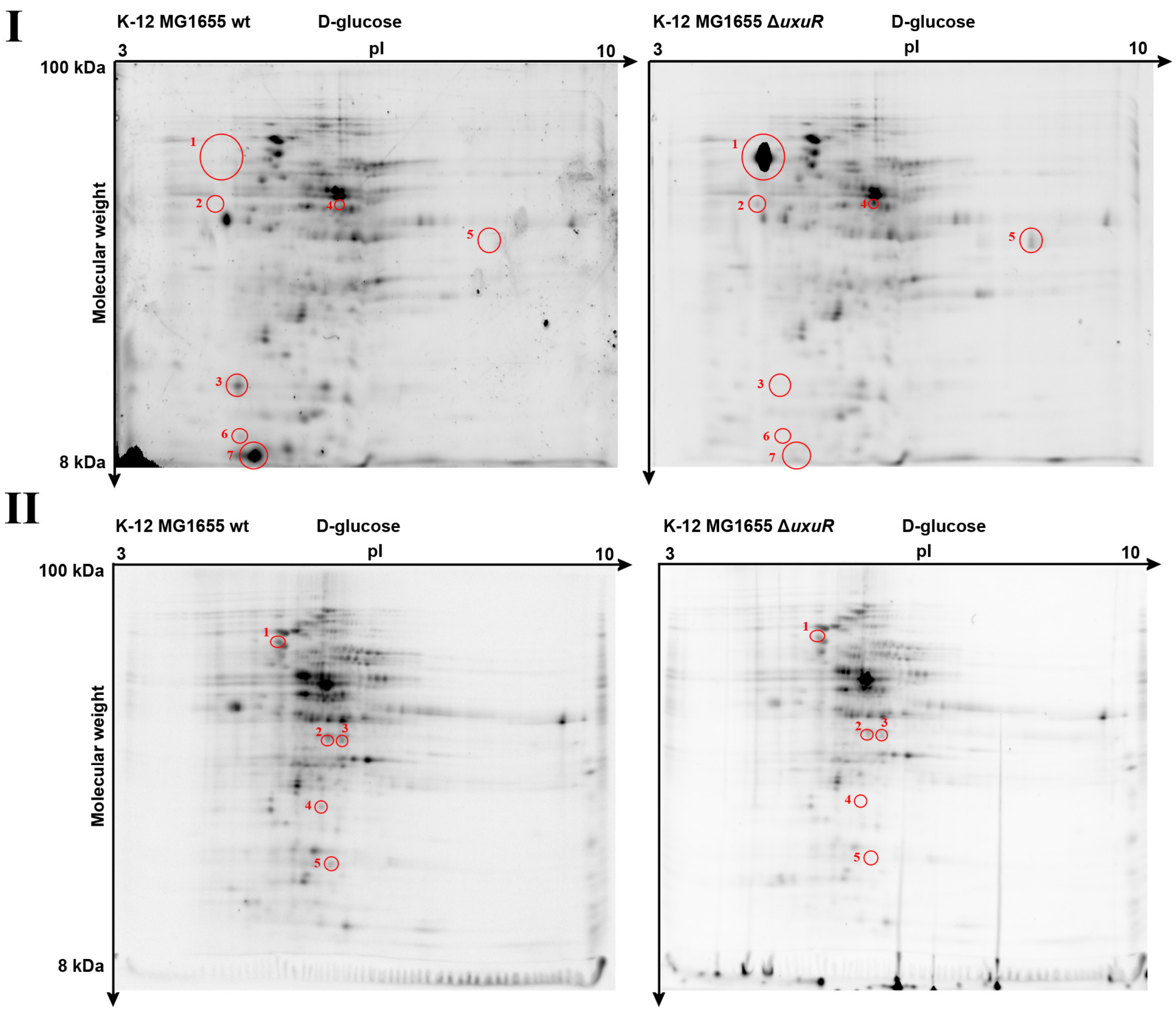

Figure S1. Proteomic map for K-12 MG1655 wild type culture growing on D-glucose or D-glucuronate. Gel cutting points for mass spectrometry are indicated as round red shapes with numbers corresponding to numbers in Table S1. I and II mean two different biological samples.

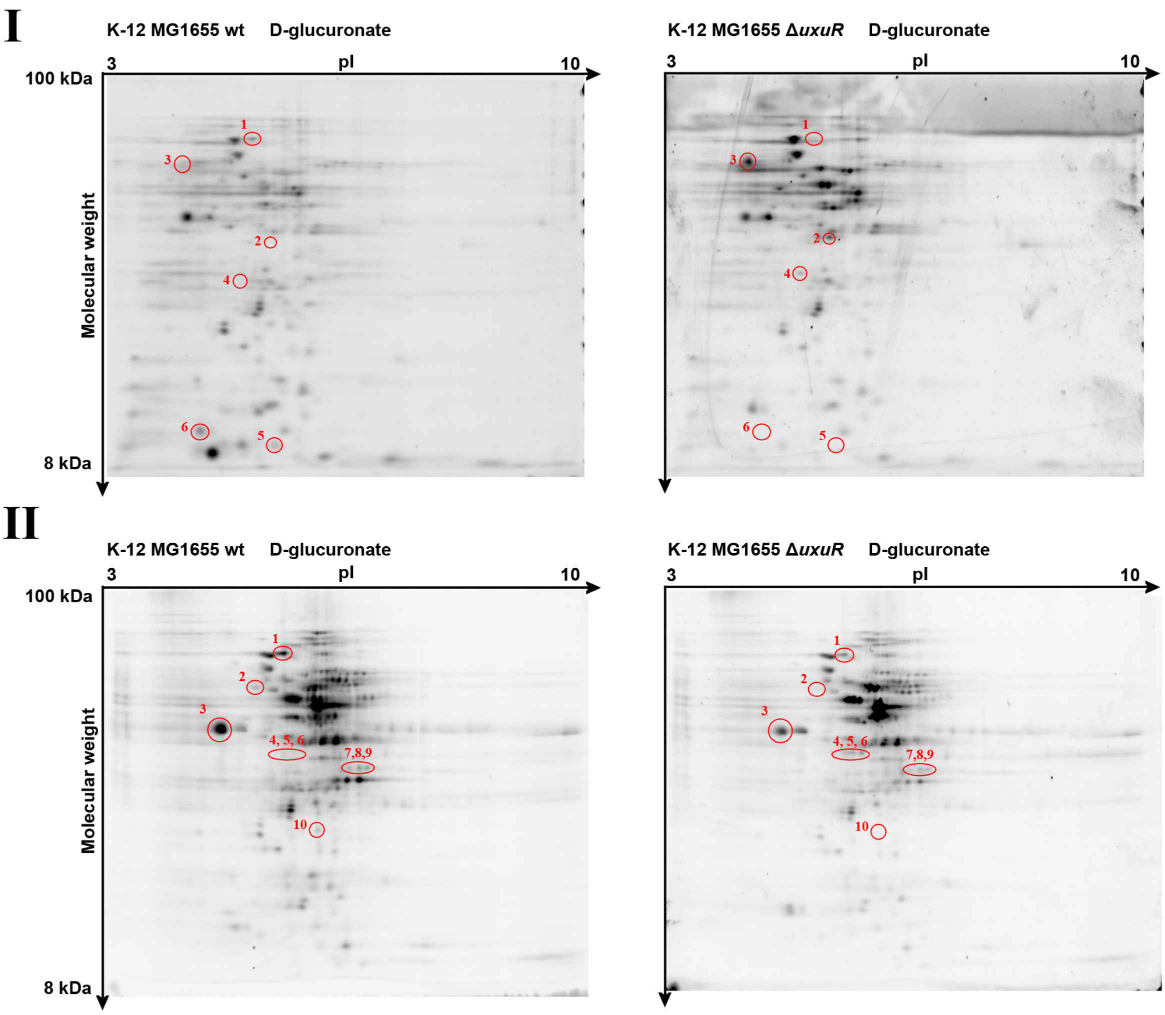

Figure S2. Proteomic map for K-12 MG1655 wild type and its Δ*uxuR* derivative growing on D-glucose. Gel cutting points for mass spectrometry are indicated as round red shapes with numbers corresponding to numbers in Table S1. I and II mean two different biological samples.

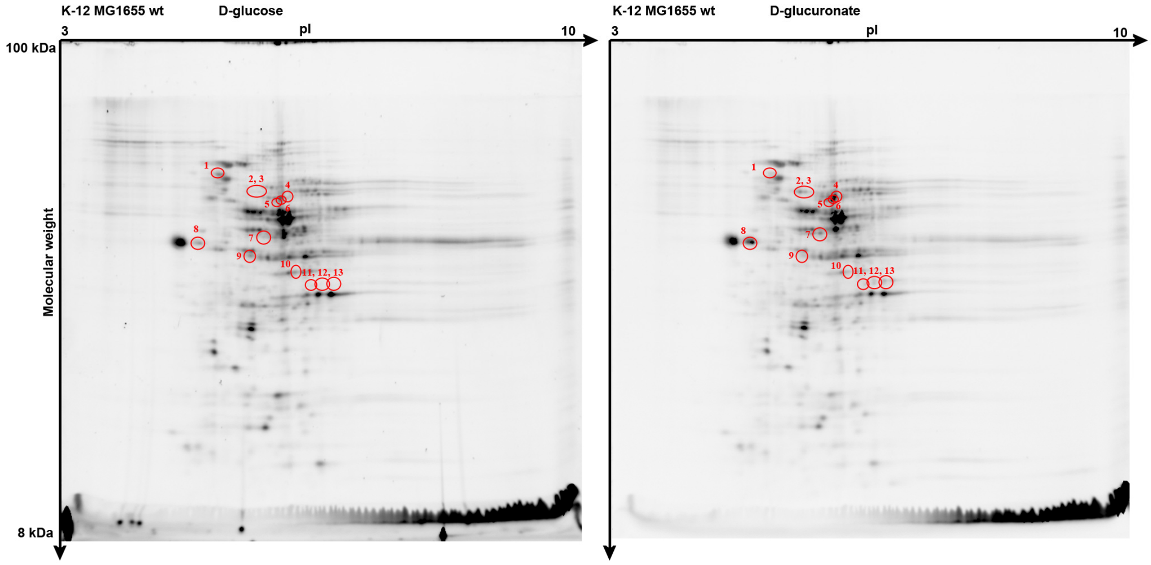

Figure S3. Proteomic map for K-12 MG1655 wild type and its Δ*uxuR* derivative growing on D-glucuronate. Gel cutting points for mass spectrometry are indicated as round red shapes with numbers corresponding to numbers in Table S1.

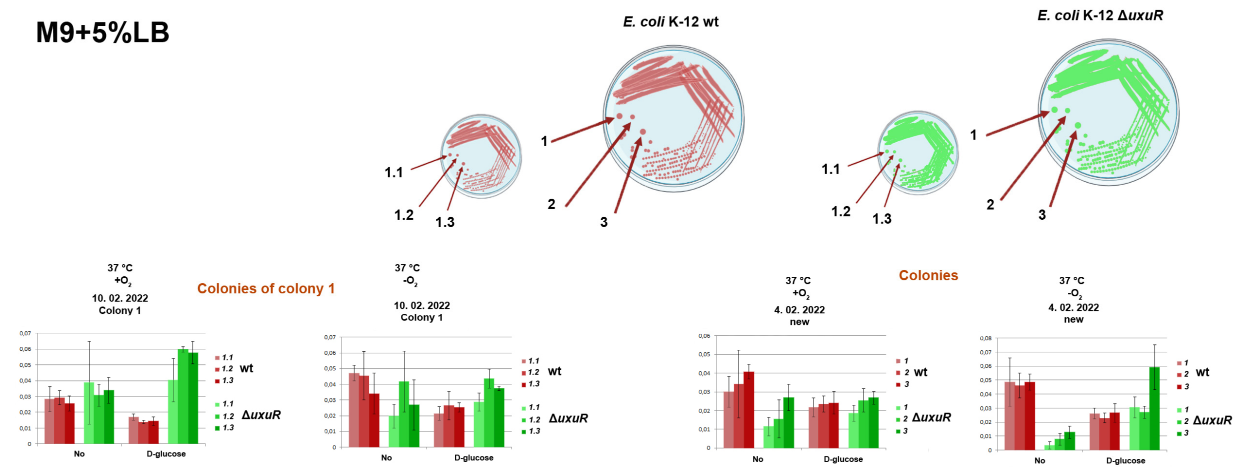

Figure S4. Series of biofilm assays for the *uxuR* deletion mutant.

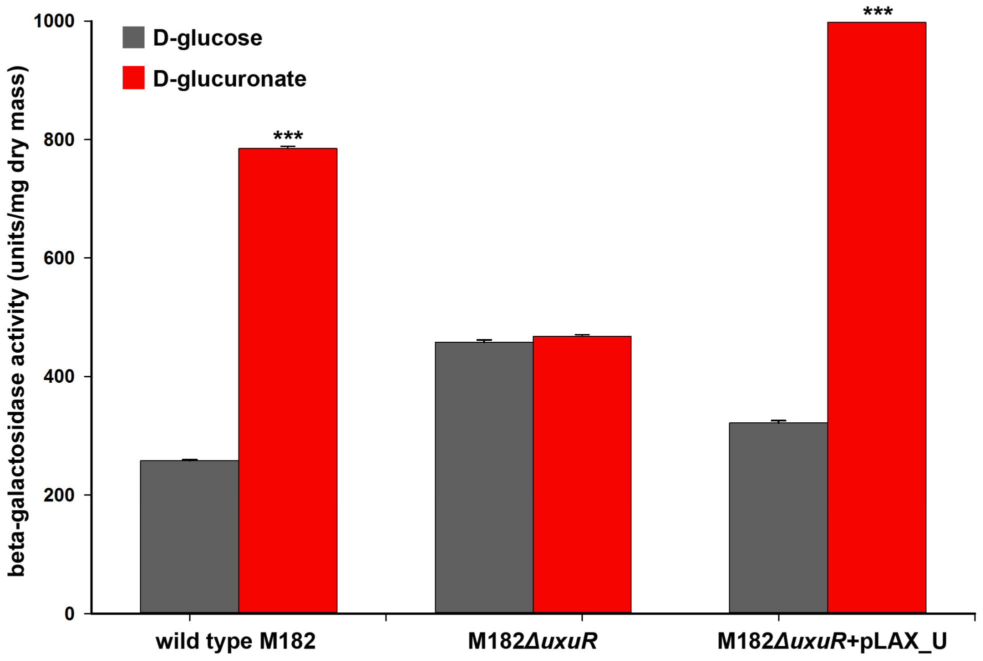

Figure S5. β-galactosidase activity under the control of *uxuAB* promoter in pRW224 in *E. coli* strain M182 with or without the deletion of *uxuR,* and with or without complementation with the pLAX_U plasmid bearing the *uxuR* gene. Error bars represent the standard deviations calculated based on three biological replicates (*** *p* value < 0.001).
